# Individual-Based Modeling Approach to Assessment of the Impacts of Landscape Complexity and Climate on Dispersion, Detectability and Fate of Incipient Medfly Populations

**DOI:** 10.3389/fphys.2017.01121

**Published:** 2018-01-09

**Authors:** Slawomir A. Lux

**Affiliations:** ^1^inSilico-IPM, Konstancin-Jeziorna, Poland; ^2^Formely: Department of Applied Entomology, Warsaw University of Life Sciences, Warsaw, Poland

**Keywords:** incipient populations, invasive propagule, trapping, pest detection, *Ceratitis capitata*, agent-based model

## Abstract

The objective of the presented study was to demonstrate the potential of a bottom-up “ethological” approach and individual-based model of Markov-like stochastic processes, employed to gain insights into the factors driving behavior and fate of the invasive propagule, which determine the initial stages of pest invasion and “cryptic” existence of the localized, ultra-low density incipient pest populations. The applied model, PESTonFARM, is driven by the parameters derived directly from the behavior and biology of the target insect species, and spatiotemporal traits of the local terrain and climate. The model projections are actively generated by behavior of the primary causative actors of the invasion processes—individual “virtual” insects—members of the initial propagules or incipient populations. Algorithms of the model were adjusted to reflect behavior and ecology of the Mediterranean fruit fly, *Ceratitis capitata*, used as a case-example in the presented study. The model was parametrized based on compiled published experimental information about *C. capitata* behavior and development, and validated using published data from dispersion and trapping studies. The model reliably simulated behavior, development and dispersion of individual members of an invasive cohort, and allowed to quantify pest establishment and detection chances in landscapes of varying spatiotemporal complexity, host availability and climates. The results support the common view that, under optimal conditions (farmland with continuous fruit availability and suitable climate), even a single propagule of medium size (100 females) usually results in pest establishment and detection within the first year post-invasion. The results demonstrate, however, that under specific sub-optimal conditions determined by the local climate, weather fluctuations and landscape topography (e.g., sub-urban), the incipient cryptic populations may occasionally continue for several generations, and remain undetected by typical pest surveillance grids for the periods extending beyond 2-years post-invasion.

## Introduction

Some of the most elusive biological phenomena—cryptic existence and fate of alien insect propagules or survival and resurgence of residual populations struggling at the verge of extinction—are actively generated by and critically depend on individual behavior of just a small number of insects, operating within environmental mosaics of the locally fluctuating opportunities, resources and threats. Such incipient populations are inherently vulnerable to stochastic uncertainties (Potapov and Rajakaruna, 2013; Rajakaruna et al., 2013) and essentially, are intractable experimentally. In fairly uniform environments, generic population models, mostly based on “random diffusion” paradigms (Rudd and Gandour, 1985; Kot et al., 2004; Liebhold and Tobin, 2008; Roques et al., 2008), largely explain the growth and spread of the initial populations, and estimate the relation between propagule pressure and probability of establishment (Memmott et al., 2005; Colautti et al., 2006; Drake and Jerde, 2009). But at the ultra-low densities and fine spatial scales of the species-typical daily exploration ranges, translocations of the individual insects are determined by the proximate configurations of environmental attributes and transient availability of resources, and thus are far from random (Lux, 2014; Manoukis et al., 2014; Lux et al., 2016, 2017). It is broadly recognized that during the initial cryptic “latency phase,” such small cohorts may linger for some time undetected (Sakai et al., 2001). But in the case of pests of economic concern, duration of the cryptic pest presence and its ultimate success—establishment or resurgence—have immense socioeconomic and regulatory ramifications (Carey, 1996; Papadopoulos et al., 2013; Mcinnis et al., 2017). Consequently, the quest for approaches and methods which could offer new insights into the mechanisms of such ephemeral processes, reveal their key behavioral and environmental drivers and quantify pest establishment and detection chances–is of the utmost practical relevance.

Confronted with the paucity of experimental options, we propose the individual-focused “ethological” approach (Lux, 2014)-stochastic simulation of lifetime events and behaviors of “virtual” individual members of the incipient cohorts, operating under hypothetical agro-ecological scenarios of varying complexity and climates. Such *in silico* emulation of the pest-landscape system offers unique possibility to capture the wealth of information about behavioral particularities of the target insect species, and reflect the impacts of the local conditions with their spatiotemporal dynamics at insect-relevant scales (An et al., 2009; Lux et al., 2016). Importantly, for very small populations scattered at ultra-low densities, such an approach permits more realistic reflection of the individual stochastic uncertainty and non-random behavioral mechanisms of the choices made in locally heterogeneous environment. Once parametrised and validated, the model permits “virtual” emulation of an unlimited number of scenarios—quantification of the net effects of even minor modifications to the topography and/or climate of the studied system (Lux et al., 2016). Such agent-based models, often in combination with cellular automata, can serve as “virtual environmental laboratories” for emulation of complex systems, and are currently used to study ecological and evolutionary processes (DeAngelis and Mooij, 2005; Jovani and Grimm, 2008; DeAngelis and Grimm, 2014), for development of environmental decision support tools (Parker et al., 2002; Parker, 2005; Grimm et al., 2014; Reed et al., 2016), modeling effects of land use and climate change (Louca et al., 2015; Hyandye and Martz, 2017), or site-specific integrated pest management (IPM) (Lux et al., 2016).

The objective of the study presented here was to demonstrate the potential of such an approach for quantification of the latency phase of “cryptic” pest existence, its detectability and establishment chances, in relation to the species-specific pest traits, the initial propagule size, the local landscape topography and climate. For this purpose, an individual-based Markov-like stochastic process model (PESTonFARM, Lux, 2014; Lux et al., 2016) was used, with its algorithms and parameters adjusted to reflect the biology and behavior of *Ceratits capitata*, a well-researched species of enormous economic importance, used here as a case-example. The potential of the presented approach and the model for site-specific assessments of the local pest establishment risks and detection chances, or for optimisation of the pest detection schemes according to the local site topography-was also discussed.

## Methods

### Outline of the model

PESTonFARM (Lux, 2014; Lux et al., 2016) is a site-focused individual-based model, which simulates behavior of individual insects operating within the locally heterogeneous environment. The model consists of two main modules, representing properties of individual “virtual” insects and traits of “virtual” local terrain.

Algorithms of the “virtual insect” module encapsulate relevant information about ecology and behavior of the target insect species, and accordingly, determine (in a stochastic sense) behavior and fate of “virtual” members of the cohorts, which represent the local pest population. Insects constitute suitable objects for such simulation, because their behavior, although intricate, can be reliably described by a set of rules linked to the traits of proximate environs. Individual behavior can be approximated by a Markov-like stochastic process, where response or fate of each insect is stochastically determined only by its current internal state and surrounding conditions (Lux, 2014). Accordingly, each behavioral step, event or “decision” of each individual “virtual” insect is fully randomized and stochastically dependent on its age, reproductive state, and on the local weather conditions, current status of the sector of its actual residence, and where relevant-also that of the nearby sectors.

Local terrain is represented by a set of matrices made by square sectors, with their values representing insect-relevant traits. The trait-values of each sector fluctuate daily according to seasonal changes in host plant phenology, host availability and infestation, local insect density and IPM treatments. Spatial resolution (sector size) is determined by pest biology and its estimated daily mobility ranges (Lux, 2014).

The model uses two key “external” forcing factors: temperature and time. The temperature represents daily average for each season day, while the time factor consists of successive season days. All phenomena are simulated with 1-day temporal resolution.

### Model adaptation to the target species: mediterranean fruit fly, *C. capitata*

The generic PESTonFARM model was developed to simulate behavior and development of frugivorous tephritid fruit flies, which share substantial similarities in their overall biology patterns. Its version 3.1 was adapted and on-farm validated for the European cherry fruit fly, *Rhagoletis cerasi*, and IPM in cherry orchards (Lux et al., 2016). For the study presented here, the model (version 4.1) was adapted and parameterized to reflect the relevant aspects of the biology and behavior of *C. capitata* (medfly). Unlike *R. cerasi*, medfly is a multivoltine species, hence accordingly, a provision was added to simulate overlapping generations and multiple 1-day-spaced discrete age-cohorts. Furthermore, the two species differ quantitatively. Thus, although most of the assumptions and processes described for the version 3.1 (*R. cerasi* model) were retained without major changes, the model was re-parameterized, based on the published information about *C. capitata* and the author's own on-farm observations. Overview of the general assumptions and simulated processes (sub-models) was provided by Lux et al. (2016), while the processes simulated in the *C. capitata* model, adopted estimates of the key parameters and their sources, are presented in Table 1.

**Table 1:** The main aspects of biology, key processes taken into account, sub-models and adopted parameters.

**Aspect**	**Process/parameter**	**Adopted values**	**Relation/sub-model**	**Basis/source**
Adult females	Sex ratio of adults emerging from the soil	1:1	Constant	Assumed
	Pattern of adult emergence from the soil	Staggered, lasting 25–40 days, 75% emerging during the first 10 days	Asymmetric bell-shaped function adjusted to fit the assumed distribution	Assumed, based on own on-farm observations
	Lifespan under constant optimal conditions (20–25°C) and in absence of extrinsic mortality causes	Aver = 79.1, max = 170 days	Gompertz function, calculated according to daily-cohort age, dynamically adjusted to account for seasonally changing temperatures	Based on: Vargas et al., 1997, 2000; Duyck and Quilici, 2002; Manrakhan and Lux, 2006; Grout and Stoltz, 2007; Carey et al., 2008; Nyamukondiwa and Terblanche, 2009
	Intrinsic mortality modeled with 1-day resolution according to individual age	1–170 days		
	Age/maturity categories, at optimal conditions (20–25°C)	1–10 Young, 11–45 Mature, 46–75 Old, 76 + Very Old		
	Extrinsic daily mortality risk caused by the complex of on-farm resident predators and natural enemies	2%	Constant	Broadly estimated, based on own and historic data
Immature stages	Sex ratio and egg status	1:1, 100% fertilized	Constant	Assumed
	Temperature-dependant duration of in-fruit development (from egg to adult emerging from the soil)	Eggs: 3–11 days, Larvae: 8–55 days, Pupae: 9–67 days	Custom-build functions, dynamically adjusted to account for seasonally changing temperatures	Based on: Duyck and Quilici, 2002; Ricalde et al., 2012
	Temperature-dependant stage survival ranges	Eggs: 60–90%, Larvae: 19–80%, Pupae: 26–70%		Based on: Ricalde et al., 2012
	Combined in-soil mortality due to extrinsic factors, e.g., predators etc.	30%	Constant	Broadly estimated, based on own and historic data
Fecundity	Mating status of mature females	Mated (100%)	Constant	Assumed
	Maximum and peak lifetime fecundity (under optimum conditions, temp. 20–25°C)	742 eggs/female, peak at 20–35 days post emergence	Custom-build function adjusted to fit the published data, dynamically adjusted to account for temperature-dependant female maturation pace	Based on: Shoukry and Hafez, 1979; Manrakhan and Lux, 2006
	Intrinsic age-dependent daily fecundity	Range: 0–16, daily individual values generated according to age, assuming normal distribution		
Mobility	Average area covered during a single local exploration errand, used to set the sector size and spatial resolution of all site-related traits	625, equivalent to 25 × 25 m sector	Constant	Assumed based on: preliminary on-farm observations
	Dispersion range	200–700 m	Not programmed, emulated by behavior of ‘virtual individuals’	Own observations, and: Meats et al., 2003
	IN/OUT balance between emigration from the modeled site/area and immigration from the neighborhood	1:0.25	Constant	Assumed for all scenarios presented in the paper
	On-site exploration, mobility, and micro-migration	Range and patterns dynamically adjusted according to female maturity, current temperature and local conditions, potential daily averages and SDs calculated according to cohort age, individual values generated based on average and SD	Custom-build age-dependent functions and algorithms adjusted to fit the published data, dynamically adjusted to account for temperature-dependant female maturation pace	Based on own on-farm observations, and: Plant and Cunningham, 1991; Meats et al., 2006; Meats and Smallridge, 2007; Navarro-Llopis et al., 2014; Pimentel et al., 2017
Host phenology, fruit suitability and infestation	Host phenology and fruit suitability for oviposition	Beginning of fruit maturation and suitability, harvest	Species/cultivar-specific	Based on: Papadopoulos et al., 2001
	Host suitability for immature development	Varied, host species specific, ranging from 60 to 100%	Constants	Assumed, partially based on own data
	Daily fruit attractiveness and suitability for larval development	Ranging from 0 to species-specific maximum	Asymmetric bell-shaped function	Assumed, function adjusted to fit data of Papadopoulos et al., 2001
	Harvest accuracy	Varied for hosts, from 40 to 80%	Constants	Assumed
	Local (sectoral) population density	Actual value for each sector		
Detection with baited traps	Trap type & density & bait type	Mc Phail trap (4/sqkm) baited with food-type (PTA) lure	Constant for all modeled scenarios and repetitions (simulation runs)	Assumed, and estimated based on experience and data of: Delrio and Zümreoglu, 1983; Lance and Gates, 1994; Kendra et al., 2010; Manoukis and Hoffman, 2014; Meats, 2014; Manoukis et al., 2015
	Trap location	Close the center of site quarter, in a host-containing sector		
	Frequency of bait change	Every 60 days	Constant	
	Daily decline in bait efficacy	0.5%daily	Constant	
	Average daily trapping risk for a newly baited trap, within the sector	5%	Constant within the sector of trap location	
	Effective bait attractiveness area, surrounding baited trap	625 sqm (25 × 25 m)	Constant, uniform within the sector of trap location	
	Responsiveness of females to baited trap	Age dependent, ranging from the initial 40 to 100% at peak, and declining to 50% when 6–7 weeks old and further down to 15% afterwards	Custom build function adjusted to fit the assumed thresholds, dynamically adjusted to account for temperature-dependant female maturation pace	Based on: Manrakhan and Lux, 2006, 2008, 2009; Meats and Edgerton, 2008; Gilchrist and Meats, 2011
Niche utilization	Fruit infestation [%]	Actual value for each sector	Custom build functions, according to type of behavior, with minor impact at low to moderate infestation level	Estimated
	Local (sectoral) daily population density	Actual value for each sector		
IPM	No IPM or any other population suppression actions was assumed	none	Relevant model functions were not activated	Assumed
External forcing factors	Time, season days	1-day resolution	constant	Assumed
	Climate (temperature)	Average daily temperature	Custom-build function, generating annual temperature patterns	Base climate approximating Crete, Greece
	Extreme weather conditions	Calm, no extreme temp. fluctuations, lack of strong winds, rain or hail storms		Assumed

### Model evaluation

The model was validated by confronting model-generated data with the results of published experiments, used as a point of reference. In all simulated hypothetical scenarios, the assumed conditions broadly resembled that in the reference studies.

General correctness of the simulation of medfly development was verified by confronting the annual population patterns generated by the model for a typical farmland landscape with the results of field experiments from Greece (Papadopoulos et al., 2001). Simulation of the patterns of fly dispersal and trapping was compared with the results of mark recapture experiments conducted by Meats and Smallridge (2007) and Plant and Cunningham (1991). For all fly dispersion scenarios, a homogenous 1 sqkm “virtual” site was generated, containing 1,600 sectors (40 × 40), 625 m^2^ each (25 × 25 m). It was assumed that the whole area is covered by uniform pattern of fruit trees, planted in a symmetric grid, with uniform canopies of moderate size (ca. 4 m diameter). Furthermore, eight traps were distributed in pairs, set at the following distances from the site center: 75, 150, 300, and 600 m (Figure 2). The traps were assumed to be baited with a lure resembling standard PTA (putrescine, trimethylamine, ammonium acetate) attractant (Ekesi et al., 2007). Simulations were conducted for three different temperature regimes, constant throughout the whole 25-week period: the optimal (25°C) and two other varied by ± 5°C (20° and 30°C). To approximate field conditions, constant 2% daily extrinsic mortality risk due to on-site resident natural enemies and pathogens was assumed, based on the author's own data. To illustrate the modulating effects of the presence of the natural enemies (extrinsic mortalities) on medfly distribution patterns, for the optimal scenario (25°C), an additional case was emulated with no extrinsic mortalities assumed. For each scenario, lifetime behavior, dispersal and trapping of a “virtual” cohort of 1,000 females were simulated. The whole cohort was “released” on day-1 from the center of the site. The virtual flies were allowed to leave the site temporarily, but no immigration was assumed to compensate for the individuals who did not return to the site during the same explorative event. For the most representative simulation from each series, weekly distribution patterns were superimposed and combined into a diagram representing density-pattern of fly presence during the cohort's lifetime. In the “reference” mark recapture experiments (Plant and Cunningham, 1991; Meats and Smallridge, 2007), only sterile insects were used, thus effectively, lifetime dispersal patterns of a single, non-reproducing adult generation were studied. Accordingly, to emulate such experiments, no reproduction was “allowed” for the released “virtual” cohort, i.e., the “oviposition” module of the model was temporarily switched off.

The results were compared with the experimental data reported in the relevant publications. The capacity of the model to reproduce the published experiments and approximate their results was treated as the evidence of correct model calibration. Afterwards, the model was “locked” and used without any further adjustments to its internal parameters.

### Virtual experiments—simulation of propagule behavior, fate and detection in landscapes of varying complexity

#### Virtual landscapes

**Figure 1:**
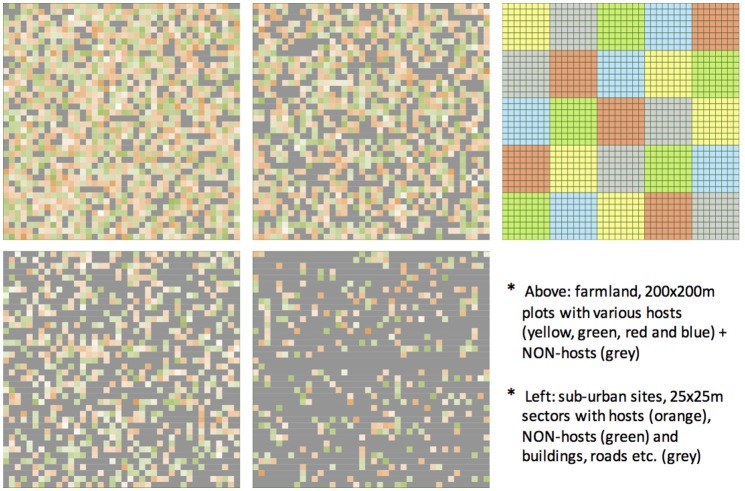
“Virtual” model-generated landscapes of varying degree of fragmentation and complexity.

Five “virtual” landscapes were generated by the model according to pre-defined parameters (Table 2), varied in the degree of their spatial heterogeneity (Figure 1). The landscapes, representing 1 sq km of terrain (further referred to as “modeled site” or “site”), comprised 1,600 square sectors, each representing 625 m^2^ (25 × 25 m) of land, arranged in 40 × 40 grid. The five types of “artificial” landscapes were designed to approximate typical scenarios, such as fruit production region (further referred to as “Farm Site”) with regular 4-hectare blocks of host and non-host trees of regular size, and four variants of peri-urban/urban landscapes (further referred to as “Urban Site 1-4”) with different density of buildings, host and non-host trees of randomly varied canopy size, land coverage and host/non-host type. The four landscapes varied in spatiotemporal complexity, fraction of land containing any trees (host or non-host) and the overall host availability, but not in the relative contribution of the host species (always 1:1:1:1) and their respective traits, such as suitability for pest development, phenology, canopy structure and quality. In the landscapes, every grid sector was individually characterized by a range of independent traits, such as: canopy coverage and size, dominant host and its traits, and daily updated about current phenology status, local pest presence, fruit infestation, trap effectiveness, IPM.

**Table 2:** Landscape structure parameters.

**Site-specific parameters**	**Farm**	**Urban**	**Urban**	**Urban**	**Urban**
	**site**	**site 1**	**site 2**	**site 3**	**site 4**
No canopy (buildings, roads)	0 (%)	20 (%)	40 (%)	60	80 (%)
Host trees	80 (%)	40 (%)	30 (%)	20 (%)	10 (%)
Non-host trees	20 (%)	40 (%)	30 (%)	20 (%)	10 (%)
**Canopy parameters (the same for or all sites)**	**Canopy coverage [%]**			**Canopy diameter [m]**	
	**Average**	**SD**		**Average**	**SD**
Host trees	50	15		4	1.5
NON-host trees	60	18		5	1.8

#### Host trees

The presence of the same four host types was assumed for each modeled scenario, jointly providing suitable fruit throughout the season. The assumption of nearly continuous fruit availability was based on experience from sub- and tropical horticulture, further supported by findings of Papadopoulos et al. (2001). The hosts varied in their phenology, overall attractiveness and suitability for development of the immature pest stages–eggs and larvae. Furthermore, for different hosts, varied harvest accuracy (the percentage of the fruit removed) was assumed, ranging from 60 to 80% (Table 3).

**Table 3:** Host and non-host parameters.

**Parameter**	**Host 1**	**Host 2**	**Host 3**	**Host 4**	**NON-host**
Relative incidence	25 [%]	25 [%]	25 [%]	25 [%]	–
Host suitability	60 [%]	70 [%]	90 [%]	80 [%]	0 [%]
Max attractiveness	70 [%]	90 [%]	100 [%]	65 [%]	29 [%]
Min attractiveness	25 [%]	29 [%]	28 [%]	23 [%]	13 [%]
Fruit availability [days]	80	65	70	85	–
Harvest time [year day]	90	170	250	350	–
Harvest accuracy [%]	65	75	80	70	–

#### Pest surveillance/detection

Each “virtual” landscape variant was “equipped” with the same “pest detection scheme.” Four pest detection traps, spaced by ca 500 m, were randomly distributed close to the center of each quarter of the modeled area (Figure 1). To emulate the usual practice in choosing the exact trap location, suitability of the potential sector was taken into account in order to facilitate the pest presence and detection, thus the trap was located in the nearest host-containing sector. The effective range of bait attractiveness was assumed to approximate sector size (625^2^ m).

#### Propagule size, invasion pattern and timing

The same invasion scenario was simulated in all cases: a single propagule with 100 females, mimicking a small number of medfly-infested fruits containing 200 larvae (male/female = 1/1) abandoned close to the center of the modeled site. To avoid undue restriction in the propagule establishment chances, the invasion was assumed to start on the 90th day of year (and of March), when the climatic and fruiting conditions were approaching its optimum.

#### Climate

Unless indicated otherwise, all simulations were conducted assuming mild Mediterranean-type climate (further referred to as “Med climate”) with the pattern approximating that of Crete, Greece (the annual min = 11°C, max = 27°C, average = 20°C). Furthermore, for selected cases, simulations were made assuming constant climate (further referred to as “Const”) with assumed constant temperature throughout the year at three levels; 20°, 25°, and 30°C, or the optimal climate (further referred to as “Opt”) with the annual temperatures following the same annual pattern as the “Med” climate, but fluctuating within more narrow, optimum range (the annual min = 20°C, max = 25°C, average = 22.9°C).

#### Conducive conditions

To avoid undue bias in emulating the cryptic phase of pest existence, and to clearly expose the effects of the propagule size, terrain structure and climate on the modeled processes, in all emulated scenarios, the following assumptions were adopted, conducive to population development:

- Continuously calm weather throughout the season, without episodes of severe winds, rain or hail storms etc.
- Nearly continuous food and host (fruit) availability within the modeled site.
- Absence of any pest suppression treatments (no IPM, except the four detection traps).
- Lack of significant Allee effect.
- Invasion start at 90th day of the year (end of March), when the temperature and fruit availability is approaching optimum.
- In the “optimal” scenario of Farm Site, the propagule arrives at central plot, which starts fruiting soon after.

#### Presentation of results

The model simulates all the processes for each grid sector and season day, and generates a wealth of detailed information. But due to peculiar feature of the incipient cohorts under the study (ultra-small size), the numbers generated daily for each sector, population densities, age structure etc., tend to be extremely erratic and thus of limited practical interest.

#### Duration of the simulated period

The simulation was conducted for 100 weeks (ca. 2 years), except the cases when the population of females present on site (all stages) reached 3,000 individuals, which was treated as a symptom of pest establishment.

#### Replications

For each landscape and invasion scenario, 15 simulations were executed, to assess development and detectability of the incipient populations founded by the invasive propagules. Each simulation was replicated with the same initialization settings. Auxiliary simulations of the selected cases were replicated 5 times.

### Statistical analysis

The results were presented as averages and either SD or 95% confidence limits. In addition, for comparison of model-generated trapping results with the published trends, a simplified process control test was used, assuming that the process is acceptably controlled (simulated), if the respective experimentally established trend points fall within 3-sigma control limits of the simulated results.

## Results

### Model validation

**Figure 2:**
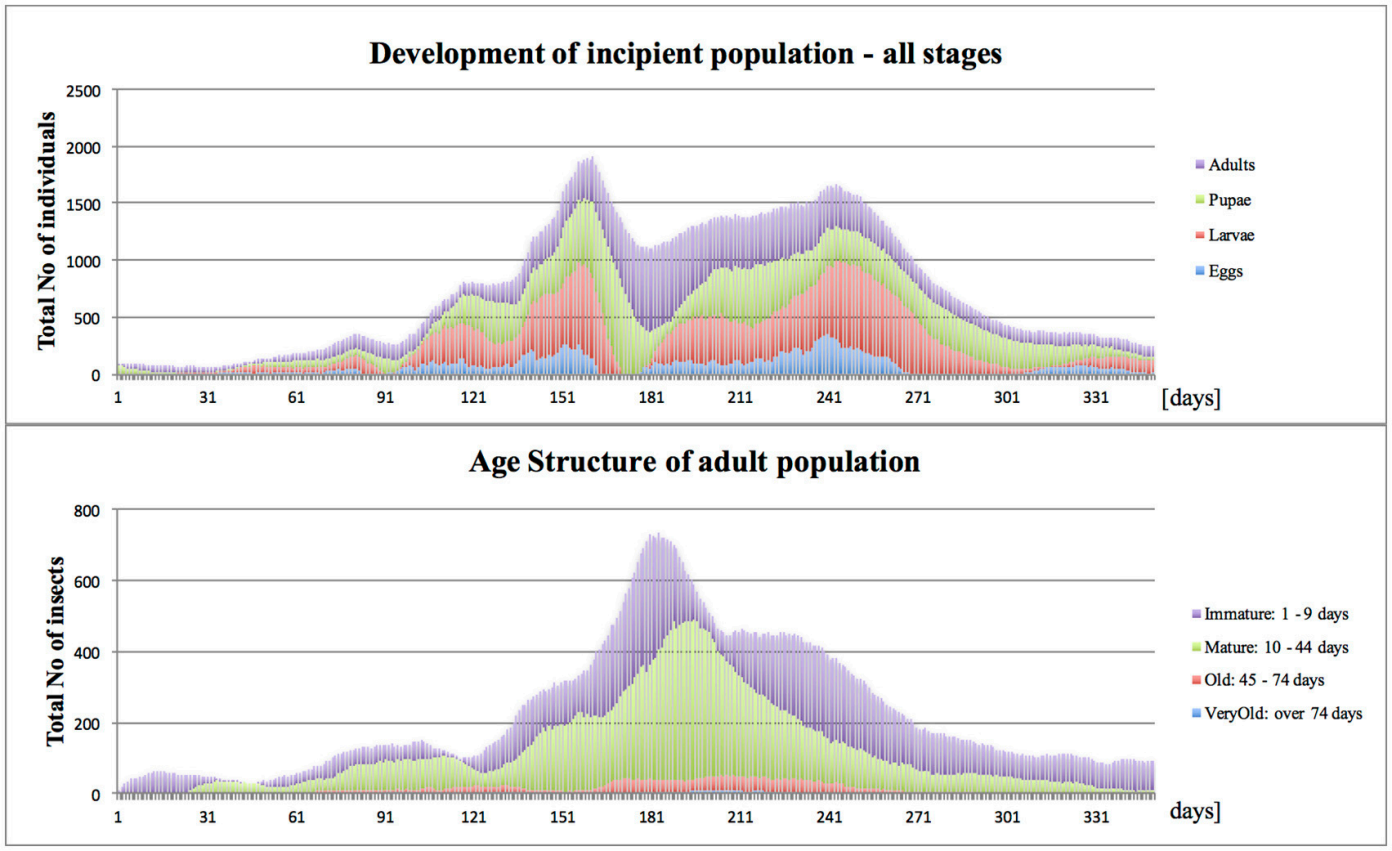
On-farm propagule development in Mediterranean climate (Start, end of March; site, Farm Site; Med climate, average = 20°C, min = 11°C, max = 27°C).

The “Mediterranean” climate (annual min = 11°C, max = 27°C, average = 20°C) broadly resembles the annual pattern of average temperatures in Crete, Greece. With brief seasons of mildly suboptimal conditions and lack of survival-threatening extremes, it is generally conducive for medlfy development throughout most of the year. Summer temperatures (reaching 27°C) only slightly reduced fly activity, and winters (10–12°C) periodically retarded or prevented fly development, and temporarily restricted or stopped their activities. During such periods, the flies remained vulnerable to mortality risks, which varied according to fly developmental stage; eggs, larvae, pupae and adults. The risks included intrinsic temperature- and age-dependent mortalities, and extrinsic ones caused by locally resident predators and pathogens. In optimal conditions (farm site with continuous host availability and lack of excessive spatial fragmentation), an invasive propagule of 100 females released in March, usually was able to establish, and within several months, substantially increase population size and reach detectable levels. The numbers of immature stages reached peak in August and to a lesser extent, also in October-November, while adults–in September (Figure 2), which resembles seasonal patterns typical for Greece (Papadopoulos et al., 2001). Also the simulated age structure of adult insects was comparable to that reported from the field (Carey et al., 2008).

**Figure 3:**
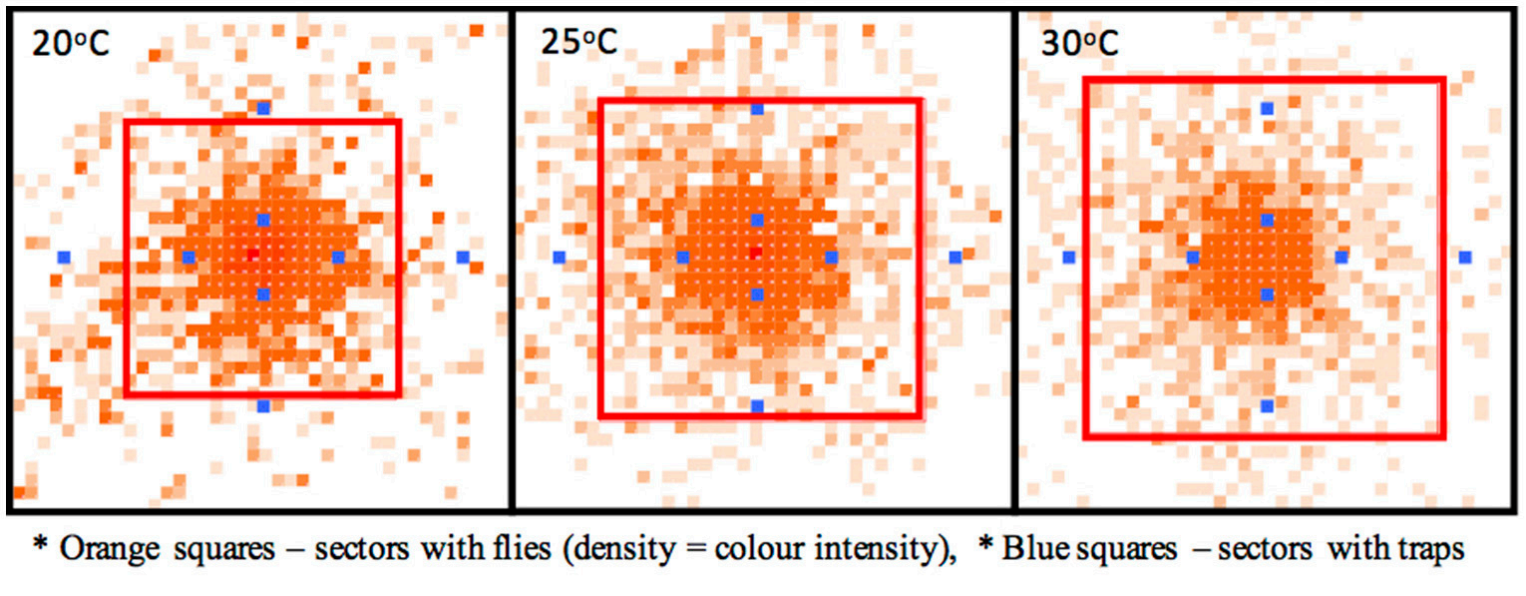
Effects of temperature on dispersion.

Lifetime patterns of fly survival, dispersal and trapping were simulated in a “virtual” homogenous orchard containing fruiting trees, and three constant temperature regimes (20°, 25°, and 30°C). A cohort of 1,000 females was synchronously released from a central point, which mimics a situation of an invasion originating from a single incident of abandoned infested fruit. Female maturation time, the average and maximum lifespan were inversely correlated with temperature (Table 4). Accordingly, the lifetime patterns of female dispersal varied with temperature as well (Figure 3). In all cases, the females distributed over the whole 1 sqkm area, with a fraction (ca. 5–13%) exploring, at least temporarily, beyond the modeled site (Table 4). The area of 90% fly “lifetime” presence was highly clustered around the release point. Its size was temperature-dependent, covering ca. 33–50 hectares (radius from 356 to 444 m) (Figure 3), and the respective 80% fly presence zones were smaller, with radius of ca. 300 m, which is in line with the experimental findings of Meats and Smallridge (2007) and Plant and Cunningham (1991).

**Table 4:** Effect of temperature on medfly dispersal, trapping and longevity^*^.

**Parameter**		**20^°^C**	**25^°^C**	**30^°^C**
90% Fly presence area [hectares]		32.7 (4.94)	45.0 (3.67)	50.5 (3.26)
90% Fly presence radius [m]		356.3 (27.95)	418.8 (17.09)	443.8 (14.00)
Exploration beyond the site [No of females]		44.8 (8.11)	124.2 (4.71)	128.0 (12.06)
Maturation time [days]		16	9	6
Aver. Longevity [days]		35.1 (1.61)	26.7 (0.48)	16.9 (0.44)
Max lifetime [days]		156.6 (12.54)	113.2 (13.74)	79.8 (6.98)
**Distance**	**Trapping (in 2 traps) and detection**			
75 m	No of trapped females	4.4 (1.14)	7.6 (2.70)	11.2 (2. 17)
	Detections/simulations^*^	5/5	5/5	5/5
150 m	No of trapped females	1.8 (0.84)	3.0 (1.00)	5.2 (3. 19)
	Detections/simulations^*^	5/5	5/5	5/5
300 m	No of trapped females	0.4 (0.89)	0.6 (0.55)	1.0 (1.00)
	Detections/simulations^*^	1/5	3/5	3/5
600 m	No of trapped females	0.0 (0.00)	0.4 (0.55)	0.6 (0.55)
	Detections/simulations^*^	0/5	2/5	3/5

* *The table contains averages of 5 simulations/replicates, and SD (provided in parentheses)*.

**Figure 4:**
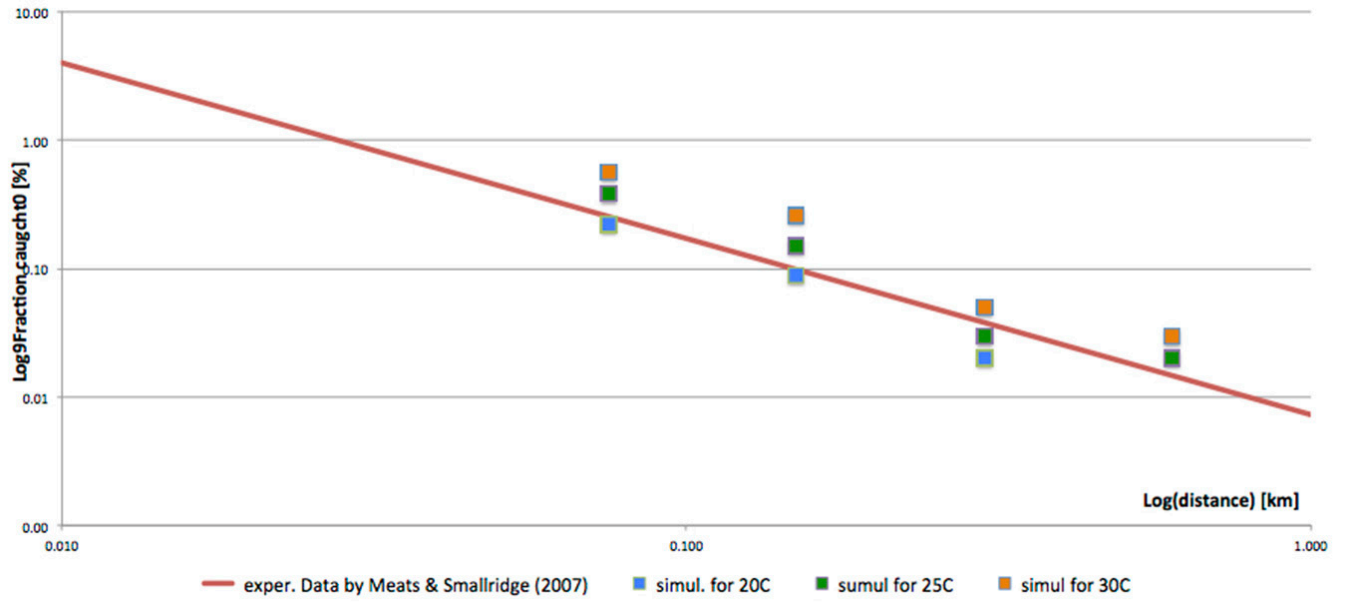
Effects of trap distance on number of females caught.

Fly catches varied strongly with trap distance from the release point, ranging from zero to 11.2 females (0–1.1% of the released cohort). In case of the traps located 75 and 150 m from the release point, the released flies were detected in each simulation. But the detection became erratic when the trap distance increased to 300–600 m (Table 4). Similar to the dispersal patterns, also trap catches and detection chances were positively correlated with temperature (Table 4). Simulated relationship between the trap distance and the fraction of the released flies trapped (Figure 4) closely fits the trends established by Meats and Smallridge (2007). The 3-sigma control limits of the simulated data points covered the respective trend points, confirming the overall correctness of the model settings and reliability of the generated data. When no extrinsic mortality was assumed (a case of laboratory conditions or a field under intense pesticide and fungicide cover), the average lifespan increased (from 26.7 to 41.8 days). In such conditions, also the 90% dispersal range increased from 45.0 to 56.3 hectares (90% radius from 418.8 to 468.8 m).

### Virtual experiments–simulation of propagule behavior, fate and detection in landscapes of varying complexity

All simulated scenarios emulated medfly invasions initiated by a single propagule of 100 females, emerging from a small number of infested fruits “abandoned” at the center of each virtual landscape.

**Figure 5:**
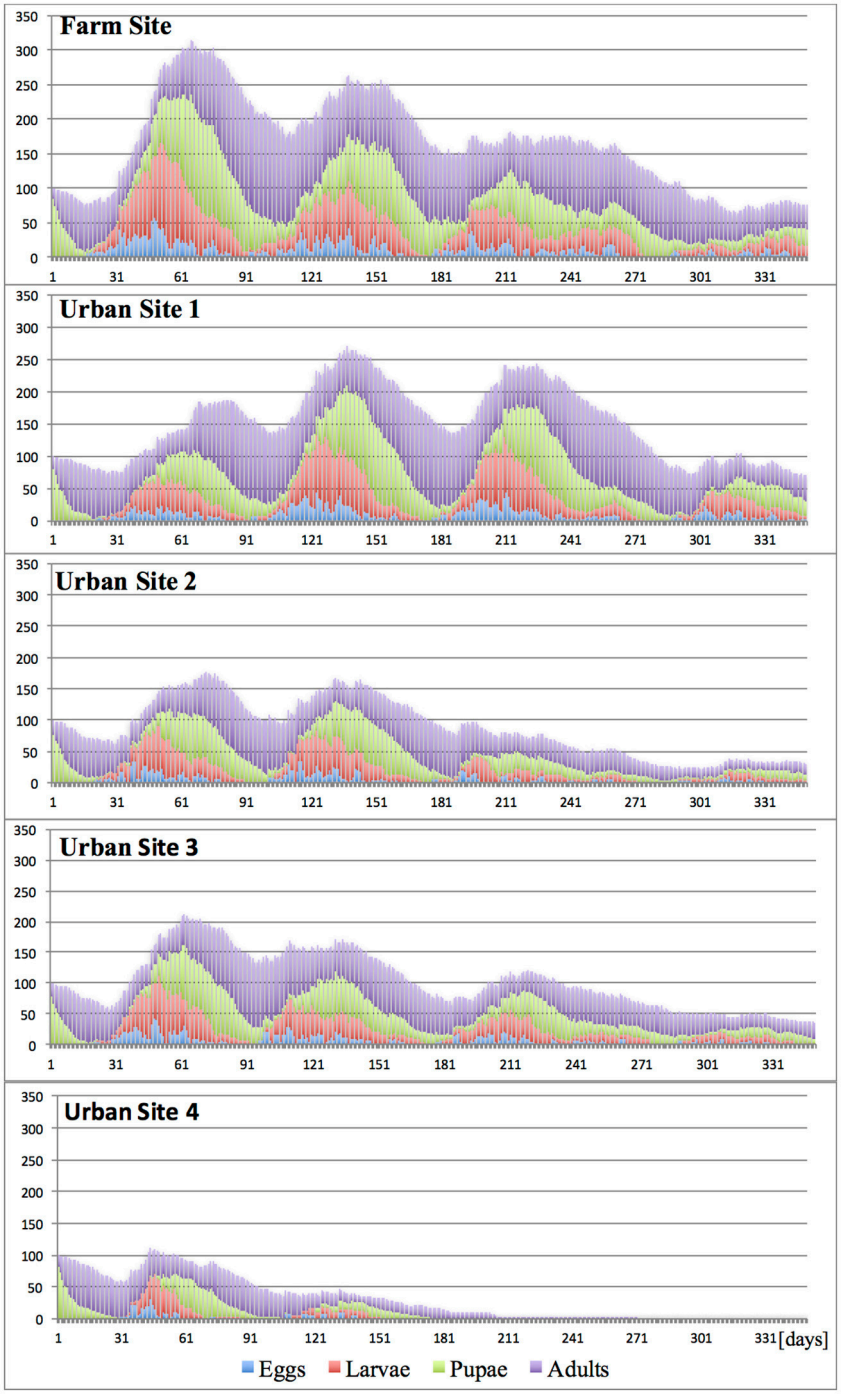
Effects of site fragmentation on propagule development at constant temperature 20°C.

**Figure 6:**
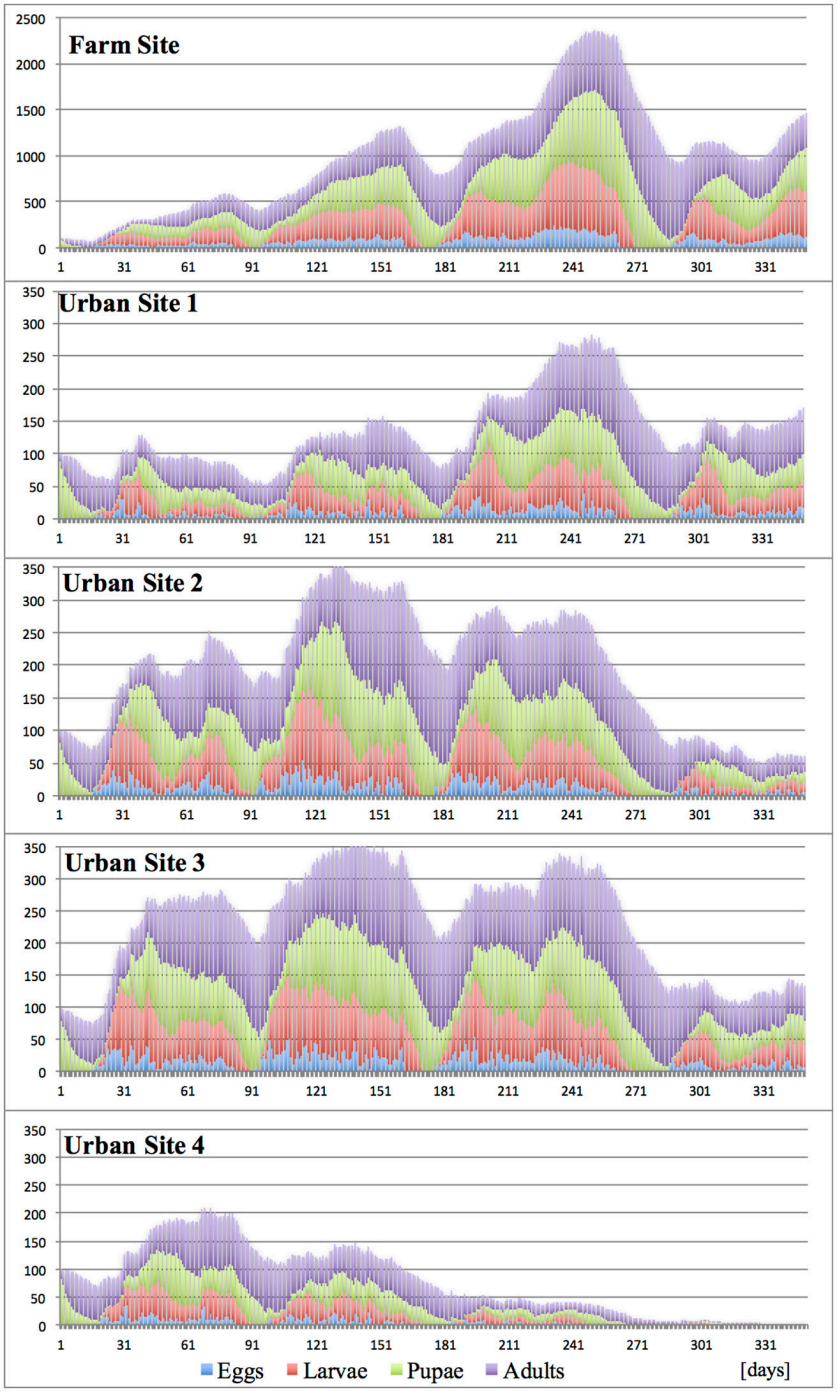
Effects of site fragmentation on propagule development at constant temperature 25°C.

**Figure 7:**
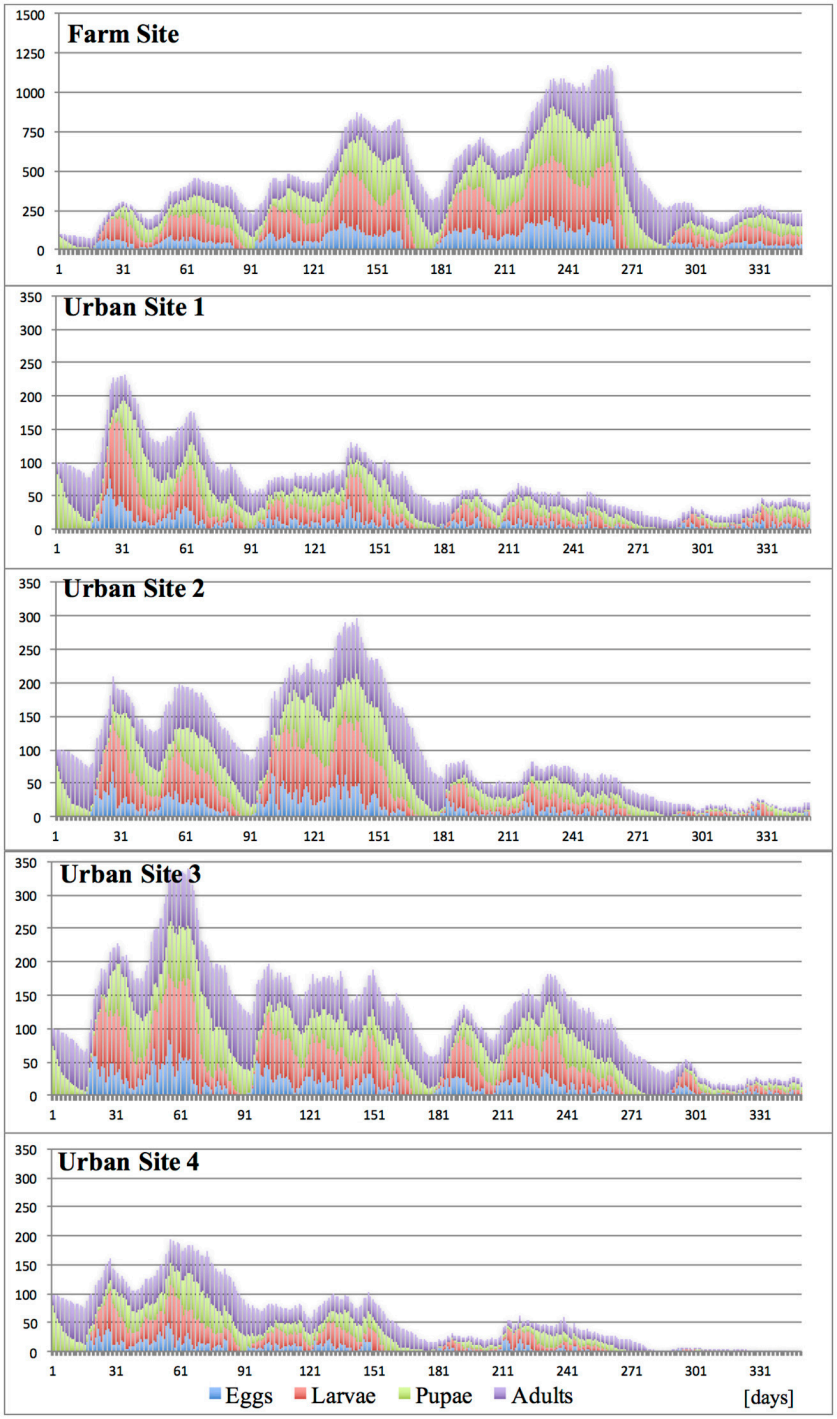
Effects of site fragmentation on propagule development at constant temperature 30°C.

*Under “Constant” climates* (20°, 25°, and 30°C)**,** with lack of any seasonal temperature fluctuation, the development of the invasive propagule was dependent on both the temperature and the degree of site fragmentation. Durations of medfly developmental stages and successive generations changed according to the temperature, and largely reflected the published data (Ricalde et al., 2012). In spite of the constant temperatures and continuous fruit availability, the host succession forcing seasonal pest shifts within the site, and variation in host traits (attractiveness, suitability and harvest accuracy) (Tables 2, 3), when combined, constituted a mild environmental factor, which jointly with different temperatures and varying site spatial complexity, substantially diversified the trajectories of propagule development and fate (Figures 5–7).

As expected, and regardless of temperature regime, the largest population growth occurred on the Farm Site comprising large blocks of adjacent diverse hosts. Maximum size of the incipient populations, reached during the first 12 months post-invasion, varied greatly between temperature regimes, and was the highest at 25°C, followed by 30° and 20°C, with ca. 2,400, 1,100, and 300 insects per site (all stages), respectively. Fragmentation of the terrain severely curtailed propagule development, and for all “Urban” sites and temperature regimes, at the peak time, the pest population ranged from ca. 100–270 insects per site. In general, in the “Urban” landscapes, the impact of temperature was less pronounced compared to the farmland, and with the exception of Urban Site 4 at 20°C, the population maxima were broadly similar. However, the population patterns and trends were not consistent, as well as the pest establishment prospects. In most “Urban” scenarios, after the initial increase, the population gradually declined, and at the end of the first year post-invasion, the numbers of surviving individuals were low, indicating unpromising pest establishment prospects. However, at 25°C, in moderately fragmented sites (Urban 1–3), the incipient populations broadly fluctuated, and at the end of the first year post-invasion, were still at the size suggesting good survival and establishment chances.

It has to be noticed, that the combined effects of temperature and landscape fragmentation were scenario-specific, non-additive and non-linear. Furthermore, these relations shall be expected to heavily rely on the local host configurations; completeness of the annual host succession chain, host traits, spatial arrangement and diversity etc., which compounds the difficulty to generalize the results.

*Under the “Optimal” climate* (annual min = 20°C, max = 25°C, average = 22.9°C) and optimal site conditions (Farm Site with continuous host availability and lack of excessive spatial fragmentation), the initial propagule quickly followed the generally expected rapid growth trajectory (Mcinnis et al., 2017). Within several months the population established, and reached the densities which, in most cases, assured detection of pest incursion.

**Figure 8:**
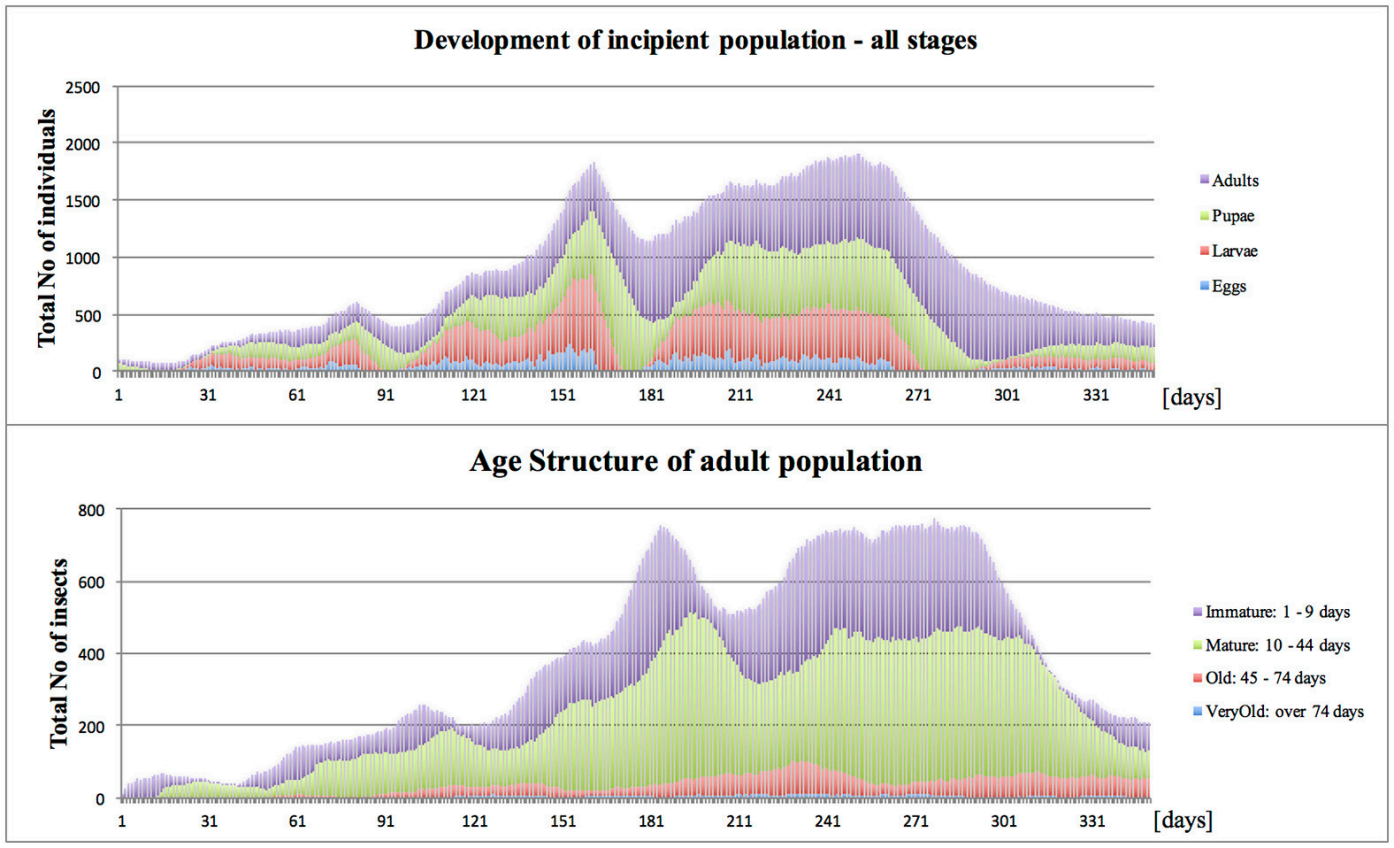
Propagule development in optimal conditions (Start, end of March; site, Farm Site; Opt climate, average = 22.9°C, min = 20°C, max = 25°C).

Relatively minor modification made to the Mediterranean climate, removal of mild barriers of cool winter and hot summer, substantially facilitated population growth. Compared to the Med case (Figure 2), under the optimal conditions (Figure 8), during subsequent generations the incipient population reached higher levels, which became the most evident toward the end of the 12-months period, which ended up with ca. 2-fold difference in the number of surviving individuals.

*Two years post-invasion*, landscape fragmentation, combined with very small propagule size and mild seasonal barriers of the “Mediterranean” climate (annual min = 11°C, max = 27°C, average = 20°C), frequently reduced the incipient populations below viability level. Barring the most extreme scenario (Urban Site 4), the chances for surviving the simulated 2 years period did not differ substantially among the landscapes, and ranged from 60 to 73%. Although generally, in the “urban” landscapes, the survival chances were inversely related to the degree of site fragmentation and host availability. Even when the incipient cohorts survived the two winters, at the end of the 2nd year simulation, the numbers of survivors were frequently below the initial propagule size, with only several individuals left at various stages (eggs, larvae, pupae, adults), which indicated bleak prospects for their future (Table 5).

**Table 5:** Propagule establishment and detection: Single 100 female cohort, emerging from centrally “abandoned fruit”^*^.

Parameter^*^		**Farm site**	**Urban site 1**	**Urban site 2**	**Urban site 3**	**Urban site 4**
Medfly survival during 2 years period (any stage) (%)		73.3	66.7	73.3	60.0	6.7
Time, if extinct (day post-invasion)		467.8 ± 86.9	480.0 ± 72.1	567.2 ± 69.4	551.4 ± 88.9	406.7 ± 46.8
Adult survival	Adult survival (%)	53.3	66.7	53.3	46.7	0.0
	No of survivors	164.0 ± 154.2	7.3 ± 2.6	4.5 ± 3.4	5.8 ± 2.9	0.0
	Maximum No during the 2 year period	286.5 ± 160.6	110.9 ± 30.2	144.5 ± 30.4	148.7 ± 31.9	75.3 ± 7.3
Immature survival	Eggs	190.4 ± 171.4	4.3 ± 4.2	0.0	4.5 ± 3.4	0.0
	Larvae	461.3 ± 426.2	15.3 ± 8.8	6.4 ± 4.1	6.6 ± 3.5	6.0
	Pupae	224.0 ± 217.6	5.9 ± 2.3	2.7 ± 1.9	2.0 ± 0.7	3.0
Detection	Detection (%)	86.7	80.0	66.6	93.3	73.3
	Day 1st detected	236.2 ± 88.8	238.3 ± 96.1	204.9 ± 31.1	130.3 ± 30.9	141.2 ± 44.9
	No trapped 1st at detection	1.0	1.0	1.0	1.0	1.0
	No of females at 1st detection	165.9 ± 108.5	57.7 ± 26.7	110.6 ± 37.2	82.6 ± 19.8	47.0 ± 11.9

* *Averages and ± 95% confidence limits, based on 15 simulations, each lasting 2 years, “Med” climate, annual min = 11°C, max = 27°C, average = 20°C*.

Remarkably, in all landscapes, the incipient cohort usually survived longer that 1 year. The average duration of extinction-ended period ranged from 407 to 567 days (Table 5), and usually, was terminated during the second winter. As to be expected, the chances for successful pest establishment were the highest on a “farmland” with solid blocks of host trees (Table 5), where after 2 years, the number of on-site residing insects (all stages) increased ca. 10 times, confirming pest establishment. Although even there, it varied, depending whether the initial propagule arrived on a plot containing host trees fruiting soon after the arrival, or on a host bearing fruits later in the season. In the latter case, the establishment chances and the number of insects present on a farm at the end of the 2 year period were lower, and the instances of extinction happened earlier.

Precise estimation of pest detection chances was not our objective; nevertheless, numerous non-detection cases, which occurred within the first 2-years post-invasion (detectability ranging from 66 to 93%), reveal that the detection process of small incipient populations is erratic (Table 5). On average, the detection happened ca. 4–8 months post-invasion, thus generally, the first few invading generations usually developed undetected. Interestingly, in the “Urban” sites, the relation between the pest detection chances and the degree of landscape fragmentation was not straightforward. In the most fragmented sites (Urban Site 3 and 4), the pest detection, if occurred, seemed to happen earlier than in the farmland. Furthermore, in the moderately fragmented Site 3, the actual chances for pest detection were the highest (Table 5). The latter indicates that a degree of landscape fragmentation may lead to concentration of the insects in fewer spots, which might actually facilitate pest detection. In general, however, with such low density incipient populations, the actual detection seems largely stochastic, which finally tends to occur after sufficiently long time. On many occasions, the detection happened well after the incipient population reached its peak, often at the time of its decline, when very low numbers (ca. ca. 40–60) of insects were actually present on farm (Table 5). The results demonstrate, that in urban scenarios, quite frequently (ca 35% of cases) such incipient “cryptic” populations may remain undetected for prolonged time, at least as long as 2 years.

## Discussion

The objective of the presented study was to demonstrate the potential of individual-based stochastic process emulation model, driven by the parameters derived directly from biology of the target pest species, and spatiotemporal representation of the local terrain and climate. The paper represents bottom-up “ethological” approach employed to gain insights into the factors driving the initial stages of pest invasion or “cryptic” existence of incipient pest populations. It is focused on behavior of the primary causative actors of such processes, individual insects-members of the initial propagules or incipient populations. The agent-based modeling approach was chosen because it can adequately reflect the complexity and local specificity of the process, permit concurrent emulation of numerous component sub-processes determining pest behavior and development, and estimate the ultimate process outcomes.

The model, PESTonFARM (Lux, 2014; Lux et al., 2016) was adapted to broadly reflect behavioral and developmental traits of medfly, and used to emulate behavior and fate of the invasive propagules in “virtual” environments, varying in the degree of fragmentation and spatial complexity, host availability, and climatic conditions. The relevance and precision of the model depends on selection of component sub-processes, realism of the assumptions and accuracy of the input data. As may be expected with complex systems, precise quantification of all assumptions, relations and parameters is not feasible. On the other hand, including only rigorously parametrized processes, although tempting to ensure formal methodological correctness, also comes at a price (Lux et al., 2016). Discarding plausible, but only superficially quantified relations *de facto* means adopting “hidden,” and frequently much less realistic default patterns (zero-order linear relations) for the “discarded” processes. The difficulty in balancing the trade-offs between conceptual simplicity and clarity of the model, process complexity vs. our insight and data availability, model realism, generality and utility, is already recognized (Evans et al., 2014; Evans and Moustakas, 2016; Moustakas, 2017). Simple models built on generic assumptions, sometimes based on superficial analogy borrowed from other disciplines, such as random “particle like” insect dispersion, or continuous “area-wide” probability-field of an insect being caught even in a distant surveillance trap, etc. may provide sufficient approximation of large pest incursions at high population densities. For very small propagules or incipient populations at ultra-low densities, when the process outcome depends on a complex behavior and stochastically uncertain fate of a few individual insects operating in a locally complex environment, such models may still offer generic predictions which might be statistically relevant for a pool of events made of hundreds of individual invasion cases, but are likely to be of little relevance to a particular case or location.

Our model is focused on the causative actors of the early invasion process, and the complexity of their individual behavior. Hence, a pragmatic approach was taken-including into the model also putative and, in some cases, only tentatively parametrized processes, and verifying the model in confrontation with experimental data. The aim was to construct a “frame-model” for holistic emulation of the pest-landscape system at the insect-relevant scale, which could be fine-tuned once more precise information becomes available. Merits of such approach to the agent-based models were demonstrated in earlier studies (An et al., 2009; Lux et al., 2016). The same pragmatic approach was used in modeling climatic conditions. Although it is possible to use in the model hourly temperatures, the realistic gain in the precision of generated projections is usually problematic, apart from largely false “impression of the precision.” This is due to the fact that hourly temperatures are usually available from more or less distant weather stations, where the conditions and terrain are usually different. Even more importantly, in heterogeneous landscapes containing diverse tree patterns with varied canopies, the local temperatures in the spots where the individual flies actually live and operate greatly vary within the whole site, and also within individual tree canopies. Fruit flies, such as *R. cerasi*, are known for their capacity to actively seek suitable spots within the canopy, which may vary diurnally and depend on fly age (Lux et al., 2017). Because our aim was to evaluate generic scenarios, and not to provide precise pest forecasting, using average daily temperatures as the external forcing factor for the simulation process was deemed acceptable, which also provided the added benefit of increased model efficiency.

The results confirm that the model, although still tentative, generates projections closely mirroring the experimental data about temperature-dependent medfly development (Ricalde et al., 2012), age structures of field-established population and the presence of variable fractions of “old” flies, as documented by Carey et al. (2008). Also, the on-site dispersion of invasive propagules and catches by the traps located at various distances to the invasion spot-was emulated in agreement with the trends experimentally established by Meats et al. (2003, 2006), Meats and Smallridge (2007). It has to be recognized, however, that in spite of the fact that medfly is among the most intensely studied pests, our understanding of the behavior and ecology of wild populations living in complex natural conditions still remains limited and incomplete. Most of our knowledge is based on the laboratory or, to lesser extent, semi-field experiments executed in spatially homogenous agro-landscapes, mostly conducted with lab-reared flies, more often than not, originating from the colonies kept for many generations in highly artificial conditions. Although it is known that behavior of wild, locally established flies may differ compared to the newly released lab adapted insects, sometimes directions and magnitudes of such discrepancies may be difficult to estimate or, occasionally, might even contradict the “common sense” expectations. For example, the fraction of “old” individuals in wild populations in Greece was found to be much higher than previously thought, and although it is generally believed that the lab-adapted flies will live longer in lab conditions than the newly collected wild ones, in fact the residual life expectancy of the wild-trapped flies, when, maintained in optimal lab conditions, was found to be longer compared to their lab-adapted counterparts (Carey et al., 2008). Furthermore, medfly behavior, its diurnal and lifetime patterns strongly depend on the individual age, maturity and nutritious status (Manrakhan and Lux, 2006, 2008), or even conditions and scale of the experimental set-ups used during observations (Manrakhan and Lux, 2009). Even the seemingly simple phenomenon of propagule dispersion, is *de facto* a complex process-a combined outcome of several sub-processes, independently moderated by ambient temperature, individual insect age, site spatiotemporal structures etc. They include, for example varied pace of female maturation, variation in survival rates, age- and status-dependent propensities to undertake various activities, lifetime changes in the patterns of movement, etc. These seemingly unrelated processes, with inherent stochasticity components, jointly translate into individually different “intrinsic” lifespans, further curtailed by varied durations of individual exposures to extrinsic mortality factors, such as the local activity of natural enemies and pathogens. Last, but not least, distributions are modulated also by seasonally changing patterns and traits of the terrain and patterns of the local IPM practices, presence or absence of host trees, their spatial arrangement, cultivar composition and canopy structures, fluctuating phenology status etc. The resulting dispersion patterns constitute the overall outcome of all these processes. For these reasons, the models based on the random dispersion paradigm usually provide acceptable approximations for larger populations (e.g., several thousand individuals) in larger and fairly homogeneous environments, but are less adequate to study the initial ultra-low-sized cohorts (from a few to few hundred) operating at the local and highly heterogeneous scales. The presented model emulates the abovementioned sub-processes at the scales relevant to the usual ranges of medfly explorative behavior, and generates dispersion and trapping results which largely remain in line with the experience and the published experimental findings (Duyck and Quilici, 2002; Meats et al., 2003, 2006; Manrakhan and Lux, 2006, 2008, 2009; Meats and Smallridge, 2007; Carey et al., 2008; Ricalde et al., 2012).

To demonstrate the potential of the model to provide preliminary insights into general trends in medfly behavior at the early stages of invasion, fate of a 100-female propagule was emulated in “virtual,” model generated landscapes, varying in the degree of their fragmentation and complexity. In each 1 sqkm modeled site, four pest monitoring traps were located, which broadly approximates the usual trap densities employed in intense pest surveillance grids, like the ones routinely used in Australia (Meats, 2014) or California (Manoukis et al., 2014). As reported by Meats and Smallridge (2007), also in our simulations, the detection within the lifespan of the first invading generation occurred very seldom when the propagules arrived at the spot not immediately proximate to the trap. But contrary to the “common wisdom,” many times, the detection finally happened not at the time when the incipient population was at its maximum, but much later (4–9 months post-invasion), sometimes during the population decline phase, when the actual numbers of the adult flies present on site were surprisingly low (10–100 individuals/km^2^). This indicates, that in the case of ultra-small incipient populations, the detection process is highly random, related not only to the incipient population size, but largely also to the “persistence” of the trapping exercise.

As commonly expected (Mcinnis et al., 2017), the invasion into a farmland frequently resulted in rapid pest multiplication, establishment and detection. However, even in such “conducive” environment, the pace of population growth and timing of its detection was modified by a “good luck” element-whether the initial propagule arrived at the spot about to bear fruit soon, or at a plot containing a host fruiting much later in the season. In the latter case, the initial need for broad dispersion to reach the plots with suitable host substantially reduced the invading cohort and retarded the population growth. Alike, in the climates with seasonally suboptimal conditions (cool winter or very hot/dry summers), the timing of the invasion largely determines the prospects for propagule development and establishment.

**Figure 9:**
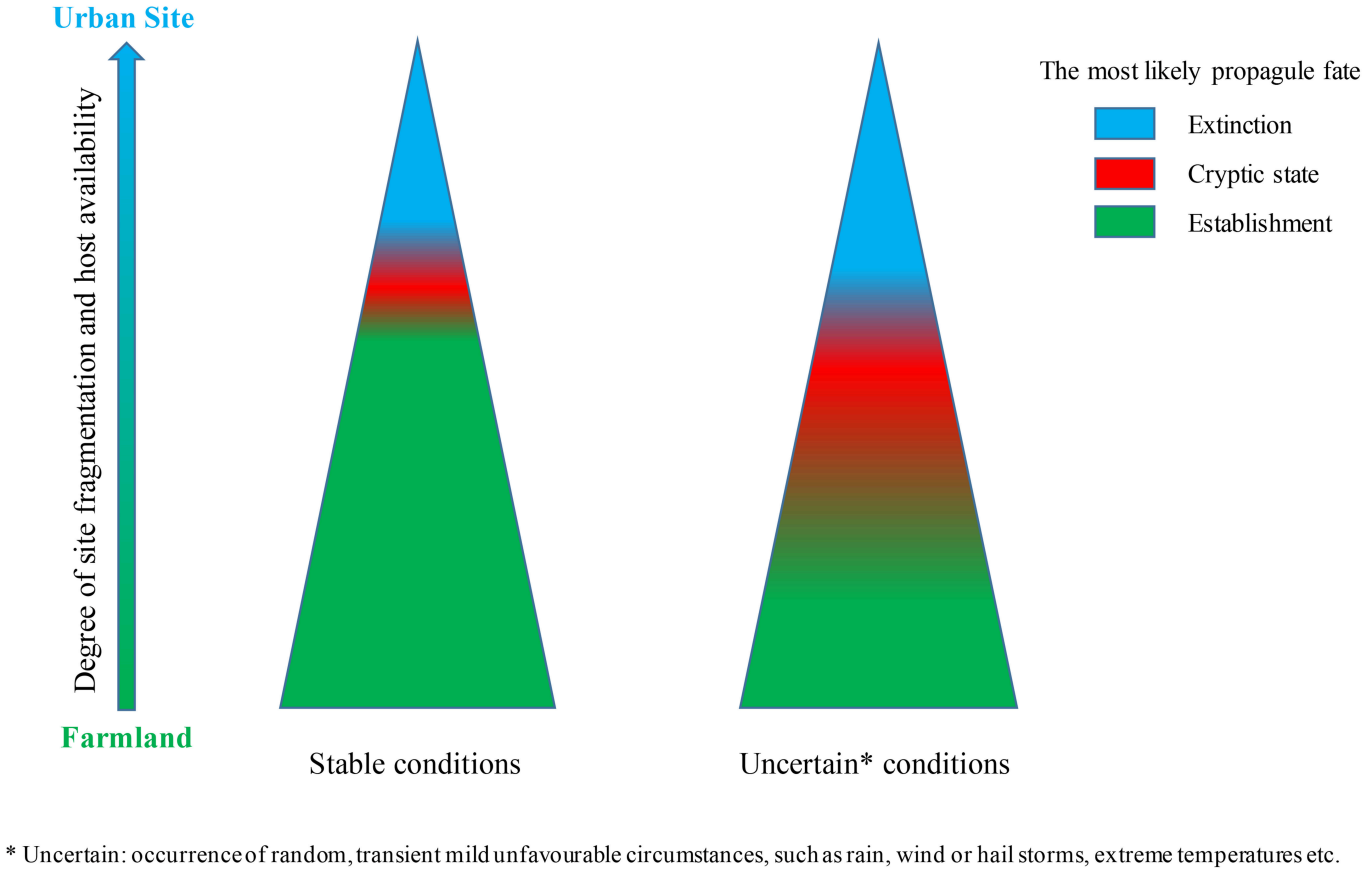
Occurrence of medfly “cryptic” phase under periodically suboptimal conditions of varying uncertainty.

In more fragmented landscapes, broadly representing various sub-urban scenarios, the processes of pest establishment and detection become progressively more erratic along with the increase in site fragmentation and complexity. In many cases, the detection occurred after more than a year post-invasion, and on several occasions failed during the whole 2 year-long modeled period, despite continuous pest presence in the area. The set of “conducive” assumptions adopted in all reported simulations, in particular the assumed lack of significant Alee effect, propagule arrival at the pest-suitable time of the year, continuous host presence and moderate extrinsic in-the-soil mortality (30%), absence of “ephemeral” random incidences of bad weather etc., combined together, likely caused a degree of overestimation of the pest establishment and detection chances. In view of this, the obtained results, demonstrating the possibility of occasional “cryptic” existence of the pest, which can remain undetected by the pest surveillance scheme for prolonged period of a few years, gain in credibility. Schematic relations between the degree of site suitability and uncertainty and likely position and breadth of the “cryptic” pest phase are presented on Figure 9. Including into the simulations random mildly “unconducive” conditions mentioned above, in most cases broaden the “extinction zone,” but will not eliminate the “cryptic” phase, rather will increase its breadth and shift the “cryptic” zone down the “site suitability and uncertainty” continuum, presented on Figure 9. Between the two broadly recognized and largely predictable scenarios of a localized pest invasion (Mcinnis et al., 2017): rapid establishment and detection or propagule extinction (in optimal or adverse conditions, respectively), there is a “continuum of uncertainty” of variable extent, with cryptic pest presence and its erratic detection, conveniently ignored by simplistic approaches. Currently, paucity of experimental data and lack of experimental methods hamper quantitative exploration of this “uncertainty zone.” The presented approach and model, once improved, could permit to tackle this phenomenon.

Such a result is not unusual. In conservation biology, the possibility of long cryptic existence of the species considered extinct since many years and surviving undetected in small numbers—is widely recognized and well documented. On many occasions, their continuous existence in the area became revealed only by sudden “re-discovery” or resurgence caused by environmental change. Examples are abundant, and include even seemingly difficult to overlook large mammals, such as Bridled Nailtail Wallaby, “re-discovered” near Dingo, Queensland, Australia, after being thought extinct from 1937 to 1973 year (Australia Wildlife Conservancy)^1^, or Gilbert's potoroo, last sighted 1869 and, after a thorough search conducted in the 1970s, thought to be extinct, until its rediscovery in 1994 in well researched Two Peoples Bay Nature Reserve in Australia (New Scientist, 1994)^2^. Similarly, resurgence of several indigenous fish species was observed after invasion of water hyacinth at Lake Victoria in Africa (personal observation) or after overfishing the earlier introduced alien top predator, the Nile perch (Balirwa et al., 2003; Chapman et al., 2003).

Also in fruit flies, in the areas with well-established and broadly spread medfly populations (e.g., in Greece), seasonal interruptions in our capacity to monitor or detect the pest presence, either by fruit sampling or trapping, are well known from the experience and are documented in the literature (Papadopoulos et al., 2001). In fact, such situations are fairly typical for the areas with regular periods of suboptimal climatic conditions, hampering insect development, reproductive and explorative activities, and temporarily reducing their responsiveness to attractants. During such seasons, the pest population is also periodically decimated to very low or residual levels, which, upon the onset of more favorable conditions, especially in farmland landscapes with sufficient continuity of host presence, rapidly builds up again and reaches easily detectable levels.

Very small invasive propagules, founding highly localized incipient populations, present more complex case. Their precarious existence is threatened by even broader range of adverse factors, which can push them into the peril of extinction (non-establishment) or maintain for some time at low levels. The possibility for substantial delays in detecting such point-source invasions even in favorable conditions, extending beyond generation time, was already reported (Meats et al., 2003; Mcinnis et al., 2017). Favorable incidents, such as transient suppression of the local natural enemies, local alteration of soil cover temporarily reducing mortality of the flies emerging from soil, or random availability of “un-harvested” suitable fruit source, permit such incipient populations, lingering at the verge of extinction, to expand and reach detectable and economically concerning levels. On the other hand, seasonally occurring mild suboptimal conditions combined with landscape fragmentation periodically restrict the cohort size, and extend the cryptic (undetected) period of pest presence. The latter could be extended much further, if the already small local cohort becomes randomly attenuated by occasional spells of unfavorable weather, even short breaks in the local annual “fruit-chain,” more accurate local harvests or IPM treatments etc. Such randomly occurring events, which nevertheless might constitute typical feature of some locations, will repeatedly inhibit the population growths or reduce it back close to the starting levels, maintaining the “fluctuating equilibrium” state of the incipient cohort, thus substantially extending the duration of its “cryptic” phase.

In addition to the fundamental and scientific merits of exploring this elusive biological phenomenon, in the case of medfly, the question of realistically plausible durations of the “cryptic” existence of undetected incipient populations has immense socioeconomic and regulatory ramifications, leading to controversy in estimations of its extent. It is generally accepted that under favorable host and climatic conditions the invading flies will either not survive or will reproduce rapidly, and thus are likely to be detected within the first few generations (Mcinnis et al., 2017; Shelly et al., 2017), which was also demonstrated by our simulations. However, the possibility that such populations could remain undetected for more extended periods (years) is widely questioned (Mcinnis et al., 2017; Shelly et al., 2017). Consequently, recurrent pest detections in the areas deemed pest-free are then attributed to random new invasion incidents, further justified by analysis of trade, commodity and human movements (Szyniszewska, 2013). On the other hand, the documented pest detections in largely the same (or similar) and, frequently, sub-urban environments, recorded at stochastically similar intervals ranging several years, provide grounds for the opinion about the possibility of longer durations of the “cryptic” pest phase (Carey, 1996; Papadopoulos et al., 2013; Carey et al., 2017), stretching beyond the already accepted periods of a few generations (Mcinnis et al., 2017). Because of the inherent experimental “intractability” of the incipient populations existing at the ultra-low densities, both the opinions postulating the occurrence of several-year-long cryptic phase, as well as the views negating such possibility-are largely based on indirect evidence.

In this context, it has to be emphasized that the presented results do not allege to represent any “real” situation in any particular country/location, or discuss regulatory implications of the biological phenomenon of variable durations of the “cryptic” pest phase. The results illustrate hypothetical scenarios and “general” relations between the fate of invasive pest propagule, landscape topography and climate, and demonstrate the capacity of the proposed approach to comprehensively emulate the relevant processes and quantify their outcomes. The presented stochastic individual-based model, based on strictly “insect-focused” approach, has the capacity to offer plausible projections, solely based on experimentally documented knowledge about individual pest behavior and the relevant traits of the local environment. The presented results demonstrate, that such approach offers new, pest-biology-based insights into the details of this elusive process.

To progress beyond this “potential-demonstration” stage, several information gaps about medfly ecology shall be filled, in order to substitute some of the experience-based estimates used in the model with more accurate experiment-based data. As demonstrated earlier in the case of the European cherry fruit fly*, R. cerasi*, the model, when parametrised to reflect “real” local site topographies, IPM conditions and climates, and tuned to emulate specific behavioral traits of the locally-acclimated on-site residing insects, can generate fairly realistic and accurate assessments of diverse site-specific scenarios and provide guidance for the local IPM improvement (Lux et al., 2016). Thus, potentially, the model could serve to identify important gaps in our knowledge about the ecology and behavior of incipient fruit fly populations, estimate impacts of various environmental traits and topography arrangements, and possibly, exploit the local spatiotemporal landscape heterogeneity for enhancement of the local pest surveillance schemes.

## Conclusions

1. The model reliably simulates propagule behavior and development, its fate and detection in landscapes of varying spatiotemporal complexity, host availability and climates.
2. The results support the common view that, under optimal conditions (farmland with continuous fruit availability and suitable climate), even a single propagule of medium size (100 females) usually results in pest establishment and detection within the first year post-invasion.
3. The results demonstrate, however, that under specific sub-optimal conditions determined by the local climate and landscape topography (e.g., sub-urban), the incipient phase may occasionally continue for generations and stretch beyond 2-years post-invasion, being undetected by typical pest surveillance grids.

## Author contributions

SL conceived the project, contributed proprietary generic PESTonFARM model, made all model adaptations (conceptual modifications, algorithm & code writing, assumptions & calibrations), executed and interpreted all the simulations used in the manuscript, and wrote the manuscript.

### Conflict of interest statement

The author declares that the research was conducted in the absence of any commercial or financial relationships that could be construed as a potential conflict of interest.

## Footnotes

^1^http://www.australianwildlife.org/wildlife/bridled-nailtail-wallaby.aspx Accessed March 14, 2017

^2^https://www.newscientist.com/article/mg14419562-300-potoroos-return/ Accessed March 14, 2017
